# Supplementary material for: Depletion of Mitochondrial Components from Extracellular Vesicles Secreted from Astrocytes in a Mouse Model of Fragile X Syndrome
Source: Int J Mol Sci. 2021 Jan 2;22(1):410. doi: 10.3390/ijms22010410 (PMC7794859; doi:10.3390/ijms22010410)
Supplement: Supplementary file 1 [file ijms-22-00410-s001.pdf]

Supplementary Materials

Supplementary materials can be found at www.mdpi.com/xxx/s1.

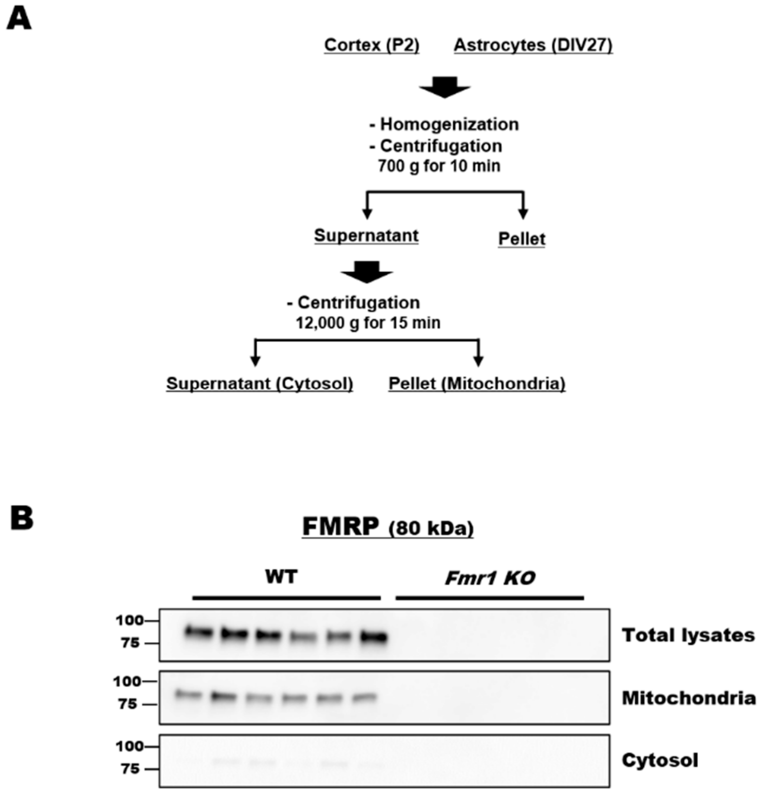

Figure S1: Mitochondrial fractionation. (**A**) Diagram of mitochondrial fractionation process. (**B**) Fragile X mental retardation protein (FMRP) is detected in the total brain lysates and mitochondrial fractions from wild-type (WT) but not Fmr1 KO mice (n = 6).

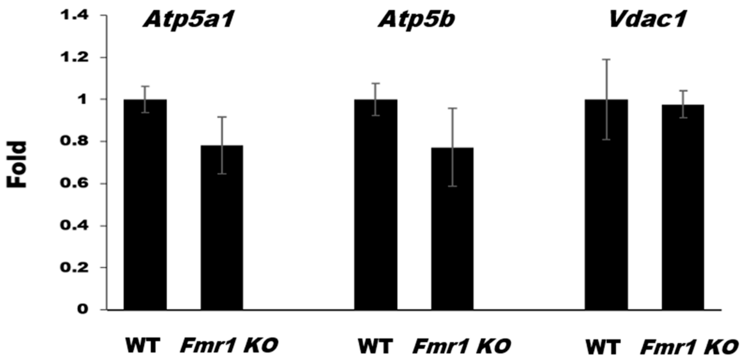

Figure S2: The relative expression levels of Atp5a1, Atp5b, and Vdac1 in the cortex of WT and Fmr1 KO mice. Each value represents the mean ± SD (n = 6 per group).

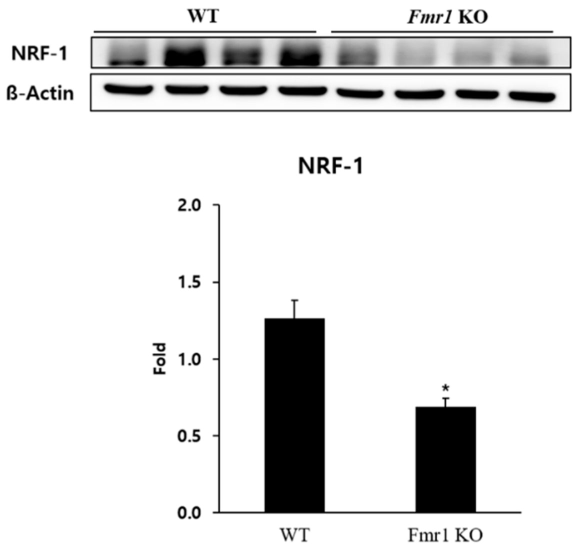

Figure S3: The relative expression levels of NRF-1 in the cortex of 10 weeks old WT and Fmr1 KO mice. Each value represents the mean ± SD (n = 4 per group).). **p* < 0.05 vs. WT mice.

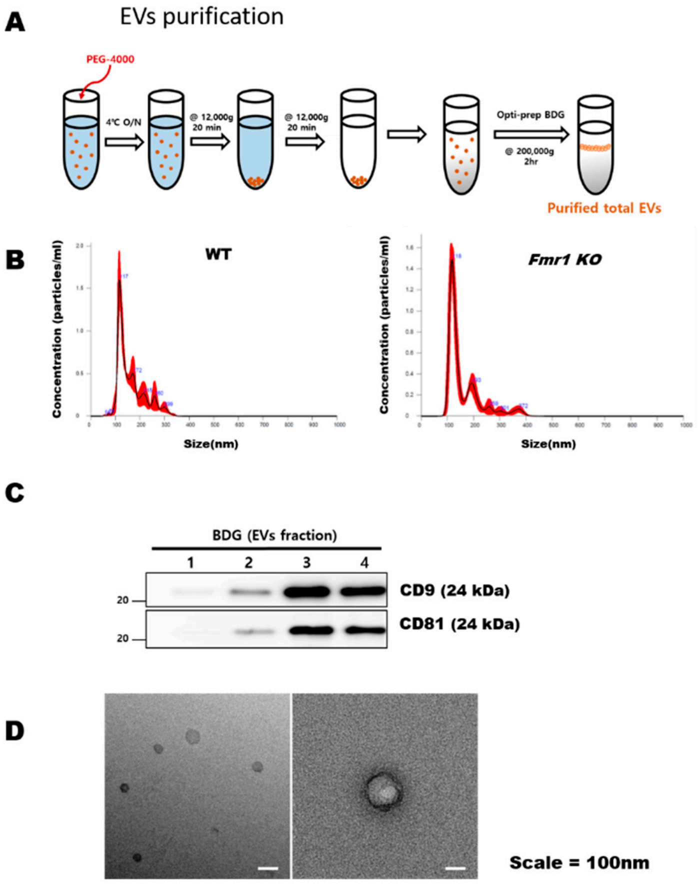

Figure S4: Isolation and purification of EVs. (**A**) Schematic illustration of buoyant density gradient ultracentrifugation for EVs isolation. (**B**) Western blot analysis of gradient fraction for the EVs markers CD81 and CD9. (**C**) Nanoparticle tracking analysis (NTA) of EVs fraction #3. (**D**) Transmission electron microscopy analysis shows the vesicles with lipid bilayer. Scale bars, 100nm.

Table S1: Sequences of primers for qRT-PCR.

Gene	Sequence (5′ →3′)	
	**Forward**	**Reverse**
Nfe2l1	GCCGCATGTGTTTGAGTCTA	CACTCGCGTCGTGTACTCAT
Nfe2l2	GAGATGAGCTTAGGGCAAAAG	TGCTCCTTGGACATCATTTC
Tfam	CCAGCATGGGTAGCTATCC	AGTTTTGCATCTGGGTGTTTAG
p0	GCACTTTCGCTTTCTGGAGGGTGT	TGACTTGGTTGCTTTGGCGGGATT
16S	CGCAAGGGAAAGATGAAAGAC	TCGTTTGGTTTCGGGGTTTCG
mt-Co1	TCTACTATTCGGAGCCTGAG	CTACTGATGCTCCTGCATGG
Atp5a1	GTCTCTCCGAGAAGCTGCAAG	AGTCGAGTGTTAGAGGCATGG
Atp5b	AGATGAACGAACCACCTGGC	GCCTGGGTAAAGCGGAAGAT
Vdac1	TGGGGTGGTTTTCTTTCTCCTCC	CTAGTGTTGTCAGGGTGTGACC
β-actin	TCTGGCACCACACCTTCTAC	TTTTCACGGTTGGCCTTAG

Table S2: Antibodies for mitochondrial biogenesis proteins

Company	Catalog no.	Name	MW (kDa)
Abcam	ab175932	Anti-NRF1 antibody	54
Abcam	ab14705	Anti-MT-CO1 antibody	40
Abcam	ab14748	Anti-ATP5A antibody	53
Abcam	ab14734	Anti-VDAC1 / Porin antibody	31
Abcam	ab14730	Anti-ATPB antibody	52
Abcam	ab138351	Anti-mtTFA antibody	29
Abcam	ab33697	Anti-TAPA1 (CD81) antibody *	24
BD Bioscience	553758	Anti-CD9 antibody *	24
Abcam	ab7260	Anti-GFAP antibody	55
Thermo Fisher	MA5-11883	Anti-Vimentin antibody (V9)	54
Cell Signaling Technology	4317S	Anti-FMRP antibody	80

* CD81 and CD9 require nonreducing electrophoresis for western blotting.
